# Measuring and decomposing oral health inequalities in an UK population

**DOI:** 10.1111/cdoe.12071

**Published:** 2013-08-31

**Authors:** Jing Shen, John Wildman, Jimmy Steele

**Affiliations:** 1 Institute of Health & Society, Newcastle UniversityNewcastle Upon Tyne, UK; 2 Economics and Institute of Health & Society, Newcastle UniversityNewcastle Upon Tyne, UK; 3 School of Dental Sciences and Institute of Health & Society, Newcastle UniversityNewcastle Upon Tyne, UK

**Keywords:** concentration index, decomposition, gini index, health inequality, oral health, oral health impact profile

## Abstract

**Objectives:**

With health inequalities high on the policy agenda, this study measures oral health inequalities in the UK.

**Methods:**

We compare an objective clinical measure of oral health (number of natural teeth) with a self-reported measure of the impact of oral health (the Oral Health Impact Profile, OHIP) to establish whether the type of measure affects the scale of inequality measured. Gini coefficients and Concentration Indices (CIs) are calculated with subsequent decompositions using data from the 1998 UK Adult Dental Health Survey. Because the information on OHIP is only available on dentate individuals, analyses on the number of natural teeth are conducted for two samples – the entire sample and the sample with dentate individuals only, the latter to allow direct comparison with OHIP.

**Results:**

We find considerable overall pure oral health inequalities (number of teeth: Gini = 0.68 (including edentate), Gini = 0.40 (excluding edentate); OHIP: Gini = 0.33) and income-related inequalities for both measures (number of teeth: CI = 0.35 (including edentate), CI = 0.15 (excluding edentate); OHIP: CI = 0.03), and the CI is generally higher for the number of teeth than for OHIP. There are differences across age groups, with CI increasing with age for the number of teeth (excluding edentate: 16–30 years: CI = 0.01, 65 + years: CI = 0.11; including edentate: 16–30 years: CI = 0.01, 65 + years: CI = 0.19). However, inequalities for OHIP were highest in the youngest age group (CI = 0.05). Number of teeth reflects the accumulation of damage over a lifetime, while OHIP records more immediate concerns.

**Conclusions:**

There are considerable pure oral health inequalities and income-related oral health inequalities in the UK. Using sophisticated methods to measure oral health inequality, we have been able to compare inequality in oral health with inequality in general health. The results provide a benchmark for future comparisons but also indicate that the type of health measure may be of considerable significance in how we think about and measure oral health inequalities.

Improvements in oral health that have occurred in recent decades, well illustrated by data from the UK (1), Canada and the United States (2), are important, but there remain inequalities in oral health between different socioeconomic groups. The central aim of this article is to investigate and measure oral health inequalities in the UK. Inequalities in health matter to everyone (3) and if individuals are to have the opportunity to flourish, they need to have good health (4). In this context, oral health is not different to other areas of health.

Recent research has demonstrated consistent and clear social gradients in oral health in Britain (5,6) and in other countries (2,7–9). However, sophisticated inequality measurement would seem necessary to investigate oral health inequality because socioeconomic gradients give important insights into inequalities, they do not provide measures of the scale of inequalities that are comparable across different types of health, countries or time. In the area of oral health, there has been limited use of inequality measures, notable exceptions include Listl (10), Mejia et al. (11) and Perera and Ekanayake (12), who have applied Concentration Indices (CIs) and Slope Indices of Inequality (SIIs) in their research.

To measure inequality, it is important to consider the most appropriate health outcomes. Researchers in the wider field of health inequalities often rely on self-reported health measures available in household surveys because objective measures are often too costly to collect. It is possible, however, that self-reported measures suffer from reporting error, which has implications for the measurement of inequality and the associated determinants of health (13). It is also possible that when measuring inequality, self-reported measures may behave differently to objective or clinical measures. Oral health data offer us the opportunity to compare different types of health measures, helping us to gain a richer understanding of inequalities. This article will explore the size of oral health inequalities in the United Kingdom and in doing so will examine some technical and philosophical questions of measurement using a self-reported measure of the impact of oral health on daily life and an objective clinical measure of oral health. We will use contemporary methods for measuring inequality and explore the relative contributions of the major determinants.

## Data and method

The data used in this study are from the 1998 UK Adult Dental Health Survey, comprising a representative sample of 6764 adults aged 16 and above living in private households in the UK. 6204 respondents completed the interview. Of those interviewed, 5281 respondents had some natural teeth (dentate) and the other 923 respondents had lost all of their natural teeth (edentate). Dentate respondents were given dental examination, of which 3817 completed the examination.

Two oral health indicators were used as outcome variables: the number of natural teeth and the 14-item Oral Health Impact Profile (OHIP) score. The number of natural teeth represents the accumulation of disease and damage over the lifetime. By contrast, OHIP is a self-reported measure of the day-to-day impact of oral health on the individual and comprises 14 questions in seven domains structured around Locker's model of oral health (14,15). The measure has been used widely in oral epidemiology. Here, we use a count of the number of oral health-related problems occurring fairly or very often (15), so the score ranges between 0 and 14. For this analysis, the overall score of OHIP has been reversed, so that a higher score indicates better oral health, to provide consistency with the number of teeth indicator. The information on OHIP is only available on dentate individuals, so the subsequent analysis of inequality in the number of natural teeth is carried out for two samples – the entire sample and the dentate sample, the latter allowing direct comparisons with OHIP.

We apply the Gini Index (16) and the Concentration Index (CI) (17) to measure the extent of inequality in oral health for the two outcome measures. The Gini Index and CI are based on Lorenz curves. The Lorenz curve for health is formed by plotting the cumulative proportion of health in the population against the cumulative population, ranked by health. With no inequality, this would plot a 45° line. The Gini coefficient measures the area between the Lorenz curve and the 45° line (perfect equality). A value of 0 indicates no inequality, and a value of 1 indicates perfect inequality. The CI is based on a similar procedure with the cumulative proportion of the population ranked by health replaced by the cumulative proportion of income. The Gini Index measures *pure health inequality,* whereas the CI measures *income-related health inequality*. Both measures range from 0 to the absolute value of 1. The value of 0 indicates complete equality, and the higher the value, the more unequal it is. Erreygers (18) suggests a method to adjust the indices to make them comparable across different health measures. We present both unadjusted and adjusted results.

The first step of decomposition is to run a regression model to examine social determinants of oral health, and this will help us understand oral health inequalities. The social determinants of oral health being examined in the regression analysis include income, education, marital status, social class, region, economic activities, as well as age and gender. The income variable is the log transformation of weekly household income, and social class is measured using Registrar General's Social Classification. Full details of the independent variables are listed in Table 1. A range of models have been tested before selecting the model presented.

**Table 1:** Summary statistics of outcome variables and covariants

Outcome variables	Mean (standard error)	Minimum	Maximum
Number of natural teeth *(including edentate)* (*N* = 3946)	20.93 (0.17)	0	32
Number of natural teeth *(excluding edentate)* (*N* = 3230)	24.70 (0.11)	1	32
Reversed OHIP *(excluding edentate)* (*N* = 3230)	12.30 (0.04)	0	14

		Percentage	
		
Covariants	Category	Including edentate (*N* = 3946)	*Excluding edentate* (*N* = 3230)
Gender	Male	45.06	45.85
	Female (reference)	54.94	54.15
Age group	16–30	18.42	22.45
	31–40	20.27	24.37
	41–50	17.49	20.25
	51–65	22.66	21.42
	66–95 (reference)	21.16	11.52
Education	Had some qualification	70.70	79.13
	No qualification (reference)	29.30	20.87
Marital status	Single	15.97	17.8
	Widowed	9.66	4.58
	Divorced/separated	8.08	8.30
	Married (reference)	66.29	69.32
Social class	Social class 1 professional occupations	4.28	5.02
	Social class 2 managerial and technical occupations	26.71	29.94
	Social class 3 skilled nonmanual occupations	23.77	24.64
	Social class 3 skilled manual occupations	20.17	19.1
	Social class 4 partly skilled occupations	17.61	16.13
	Social class 5 unskilled occupations (reference)	7.45	5.17
Region	England north	15.99	15.70
	England midlands	13.91	13.90
	Wales	13.38	13.31
	Scotland	19.56	17.59
	Northern Ireland	10.01	10.09
	England south (reference)	27.14	29.41
Economic activity	Part time	16.14	18.39
	Unemployed	2.33	2.63
	Retired	24.15	15.20
	Others (student, homemaker disabled, etc.)	14.12	13.81
	Full time (reference)	43.26	49.97
Income	Log continuous weekly household income	Mean: 5.82 (standard error: 0.01)	Mean: 5.94 (standard error: 0.01)

The CI is decomposed (19) to capture the linear associations between the health variable and covariants. It should not be considered as a structural model or used to infer a direction of causality. Decomposition reveals contributions of different socioeconomic factors to the income-related health inequalities. The contribution of each socioeconomic factor can take both positive and negative values. When the health variable is increasing in good health as in this case, positive (negative) CI indicates pro-rich (pro-poor) inequality, meaning inequality would decrease (increase) if the covariant was to become more equally distributed across the income distribution. All the analyses are performed using sampling weights.

## Results

Detailed summary statistics of the outcome variables and socioeconomic covariants are presented in Table 1. The mean number of natural teeth for the entire sample when edentate individuals are included is 20 (25 when edentate individuals are excluded). The average reversed OHIP score is 12, which is close to the maximum score of 14 suggesting a mean of around two oral health problems among the population. There are more female (55%) than male participants in the sample. The majority (71%) of the population have some form of qualification, and most (66%) are married. Less than half (43%) of the population is in full-time employment.

### Inequality measures

The Gini Index and CI for the entire sample, and by age and sex, are shown in Table 2. Overall, inequality is highest when oral health is measured as number of natural teeth (particularly when including edentate) and, for both pure health inequality and income-related health inequality, lowest when measured using OHIP. Gini Index and CI are also examined for each age–gender group. Pure health inequality (the Gini) increases markedly with age for the number of teeth (with and without edentate) – for both sexes combined, the Gini Index is 0.1674 in the youngest group but 0.6591 among the oldest (including edentate); however, a different trend is observed for OHIP with a slight increase up to the age group of 41–50 followed by a decrease in pure health inequality. Comparing the measures, it appears that up to age 50, inequalities for OHIP are higher than for the number of natural teeth, while after the age of 50, inequalities are lower for OHIP. For income-related health inequality as measured by CI, a similar trend is observed, although the magnitude of inequality is smaller and the age at which the relative magnitude changes is 40.

**Table 2:** Gini and Concentration Indices

Gini and CI for the whole population				

Inequality measures		Number of natural teeth (including edentate) (*N* = 3946)	Number of natural teeth (excluding edentate) (*N* = 3230)	OHIP (excluding edentate) (*N* = 3230)
Gini coefficients	Gini	0.2597	0.1267	0.0947
	Adjusted Gini	0.6796	0.4039	0.3329
Concentration Indices	CI	0.1351	0.0460	0.0088
	Adjusted CI	0.3534	0.1467	0.0309

Gini and CI for each age–gender group							

		Adjusted Gini			Adjusted CI		
			
Age–gender groups		Number of natural teeth (including edentate)	Number of natural teeth (excluding edentate)	OHIP (excluding edentate)	Number of natural teeth (including edentate)	Number of natural teeth (excluding edentate)	OHIP (excluding edentate)
16–30	All	0.1674	0.1695	0.3510	0.0121	0.0115	0.0521
	Male	0.1586	0.1657	0.3391	0.0023	0.0045	0.0430
	Female	0.1706	0.1673	0.3621	0.0205	0.0183	0.0623
31–40	All	0.2499	0.2195	0.3188	0.0651	0.0464	0.0615
	Male	0.2629	0.2272	0.2709	0.0738	0.0613	0.0409
	Female	0.2346	0.2099	0.3626	0.0579	0.0309	0.0773
41–50	All	0.3766	0.3145	0.3832	0.0973	0.0926	0.0161
	Male	0.3898	0.3107	0.3430	0.0640	0.0688	0.0324
	Female	0.3633	0.3178	0.4173	0.1269	0.1159	0.0049
51–65	All	0.7135	0.4708	0.3003	0.3083	0.1590	0.0333
	Male	0.7109	0.4749	0.2804	0.3447	0.1750	0.0353
	Female	0.7152	0.4655	0.3194	0.2615	0.1391	0.0236
66–95	All	0.6591	0.5385	0.2721	0.1861	0.1100	0.0262
	Male	0.6929	0.5719	0.2643	0.2237	0.1606	0.0061
	Female	0.6255	0.5014	0.2782	0.1390	0.0665	0.0455

### Regressions and decompositions of CI

Multiple regressions are estimated to understand the socioeconomic determinants of oral health. CI of each oral health indicator is decomposed to examine relative contributions of the covariants. Table 3 shows the regression results for all three sets of oral health outcomes. For the number of teeth, there is a clear age gradient, a social class gradient and the expected income effect. For OHIP, the results are mixed. Income has a significant impact on OHIP, but there is no clear age gradient and a less consistent and significant social class gradient.

**Table 3:** Regression results

	Number of natural teeth (including edentate)		Number of natural teeth (excluding edentate)		OHIP (excluding edentate)	
			
Covariants	Coefficient	Standard error	Coefficient	Standard error	Coefficient	Standard error
Log weekly income	1.093***	0.189	0.730***	0.139	0.271***	0.072
Male	0.087	0.259	0.067	0.194	0.148	0.100
Age group 16–30	14.969***	0.591	9.051***	0.494	−0.359	0.255
Age group 31–40	13.902***	0.567	8.364***	0.481	−0.090	0.248
Age group 41–50	11.518***	0.575	6.198***	0.485	−0.456*	0.251
Age group 51–60	5.393***	0.477	2.623***	0.424	0.015	0.219
Had some qualification	3.466***	0.304	1.738***	0.235	0.089	0.121
Single	0.098	0.337	0.256	0.242	0.227*	0.125
Widowed	−2.096***	0.473	−0.422	0.463	0.028	0.239
Separated/divorced	−0.043	0.453	0.096	0.336	−0.147	0.174
Social class 1	3.963***	0.704	2.255***	0.539	0.856***	0.278
Social class 2	3.295***	0.507	1.962***	0.409	0.327	0.211
Social class 31	2.841***	0.488	1.853***	0.399	0.515**	0.206
Social class 32	1.661***	0.504	1.425***	0.409	0.600***	0.211
Social class 4	1.481***	0.494	1.349***	0.407	0.327	0.210
North England	−2.418***	0.291	−1.293***	0.219	−0.078	0.113
England midland	−1.172***	0.302	−0.252	0.228	0.235**	0.118
Wales	−1.885***	0.525	−1.023**	0.396	0.175	0.204
Scotland	−3.673***	0.412	−1.972***	0.319	−0.070	0.165
Northern Ireland	−1.900**	0.751	−1.183**	0.557	0.339	0.288
Part time	0.637*	0.348	0.027	0.251	0.154	0.130
Unemployed	1.168	0.736	0.491	0.507	−0.441*	0.262
Retired	−0.858*	0.503	−0.899**	0.423	0.504**	0.218
Others	−0.708*	0.399	−0.460	0.291	−0.612***	0.150
_cons	2.088	1.315	11.974	1.018	10.234	0.526
*N*	3946		3230		3230	
*R* square	0.5786		0.4317		0.0453	
*F* for regression	224.36		101.45		6.34	
*P* > *F*	0.000		0.000		0.000	

* 90% significance,

** 95% significance,

*** 99% significance.

Decomposition results are presented in Table 4, with aggregated contributions calculated for each set of categorical variables. It appears that the major contribution to income-related inequality in oral health is income (following age for number of teeth outcome). For number of teeth, social class and whether respondents had qualifications make the next largest additional contributions to the overall inequality. Education is important because it has a large impact on oral health outcomes (reflected by its elasticity) and also is unevenly distributed along the income distribution (reflected by the CI for education). The latter suggests that highly educated people are over-represented at the higher end of the income distribution (demonstrated by the positive CI for education). For OHIP, the decomposition results require cautious interpretation because of the low explanatory power of the determinants of health regression. However, there is a relatively small age contribution, largely because the elasticity (responsiveness of OHIP to the covariant) of age is small. The contribution of education is also smaller. Interestingly, being retired is associated with a better OHIP score, and the retired are over-represented in the lower end of the income distribution (demonstrated by the negative CI for retired). If the retired group were more evenly distributed across the income distribution, then the measured CI (in this case) would be close to zero.

**Table 4:** Decomposition results

	Number of natural teeth (including edentate)				Number of natural teeth (excluding edentate)				OHIP (excluding edentate)			
			
Covariants	Elasticity	CI	Contr.^a^	Agg.^b^	Elasticity	CI	Contr a	Agg.^b^	Elasticity	CI	Contr.^a^	Agg.^b^
Log weekly income	0.3041	0.0805	0.0245	0.0245	0.1775	0.0748	0.0133	0.0133	0.1306	0.0745	0.0097	0.0097
Male	0.0020	0.0529	0.0001	0.0001	0.0013	0.0306	0.0000	0.0000	0.0061	0.0278	0.0002	0.0002
Age group 16–30	0.1498	0.1427	0.0214		0.0836	0.0672	0.0056		−0.0074	0.0596	0.0004	
Age group 31–40	0.1375	0.1763	0.0242		0.0839	0.0784	0.0066		−0.0018	0.0794	−0.0001	
Age group 41–50	0.0945	0.2217	0.0209	Age total	0.0516	0.1370	0.0071	Age total	−0.0073	0.1415	−0.0010	Age total
Age group 51–60	0.0548	−0.0108	−0.0006	0.0660	0.0231	−0.0146	−0.0003	0.0189	0.0002	−0.0158	0.0000	−0.0016
Has qualification	0.1189	0.1555	0.0185	0.0185	0.0566	0.1009	0.0057	0.0057	0.0057	0.1013	0.0006	0.0006
Single	0.0008	−0.0534	0.0000	Marital status total	0.0019	−0.1070	−0.0002	Marital status total	0.0036	−0.0967	−0.0004	Marital status total
Widowed	−0.0090	−0.6628	0.0060		−0.0008	−0.6410	0.0005		0.0001	−0.6644	−0.0001	
Separated/divorced	−0.0002	−0.4265	0.0001	0.0060	0.0003	−0.4504	−0.0001	0.0002	−0.0009	−0.4837	0.0004	−0.0001
Social class 1	0.0085	0.4027	0.0034		0.0047	0.3503	0.0016		0.0034	0.3593	0.0012	
Social class 2	0.0420	0.2835	0.0119		0.0242	0.2433	0.0059		0.0076	0.2561	0.0019	
Social class 31	0.0324	0.0089	0.0003	Social class total	0.0188	0.0075	0.0001	Social class total	0.0101	0.0076	0.0001	Social class total
Social class 32	0.0163	−0.1267	−0.0021		0.0112	−0.1682	−0.0019		0.0099	−0.1580	−0.0016	
Social class 4	0.0123	−0.2531	−0.0031	0.0105	0.0090	−0.2585	−0.0023	0.0035	0.0044	−0.2523	−0.0011	0.0006
North England	−0.0250	−0.0328	0.0008		−0.0084	−0.0251	0.0002		−0.0013	−0.0187	0.0000	
England midland	−0.0110	−0.1023	0.0011		−0.0014	−0.1434	0.0002		0.0036	−0.1050	−0.0004	
Wales	−0.0044	−0.0363	0.0002	Region total	−0.0056	−0.0171	0.0001	Region total	0.0007	−0.0476	0.0000	Region total
Scotland	−0.0150	−0.0524	0.0008		−0.0143	−0.0166	0.0002		−0.0005	−0.0370	0.0000	
Northern Ireland	−0.0020	−0.0770	0.0002	0.0030	−0.0049	−0.0223	0.0001	0.0009	0.0006	−0.0979	−0.0001	−0.0004
Part time	0.0049	0.1531	0.0008	Economic activity total	0.0002	0.0858	0.0000	Economic activity total	0.0022	0.0885	0.0002	Economic activity total
Unemployed	0.0014	−0.3474	−0.0005		0.0005	−0.4304	−0.0002		−0.0011	−0.3759	0.0004	
Retired	−0.0092	−0.4787	0.0044		−0.0056	−0.4529	0.0025		0.0058	−0.4856	−0.0028	
Others	−0.0043	−0.3476	0.0015	0.0062	−0.0026	−0.3605	0.0009	0.0033	−0.0065	−0.3834	0.0025	0.0003
Sum			0.1347	0.1347			0.0457	0.0457			0.0093	0.0093
Residual (total CI – sum)			0.0003				0.0003				−0.0005	
Total CI			0.1351				0.0460				0.0088	

a contributions of each individual covariant;

b aggregated contributions – sum of contributions for each set of categorical variables.

## Discussion

Our results raise a number of issues when trying to understand oral health, oral health inequalities and inequalities more generally. The most important issue, but perhaps the least surprising, is that measurable oral health inequalities exist and can be significant. There is clear evidence from this work that individuals at the lower end of the income distribution suffer from worse oral health than individuals at the higher end.

Given the nature of the conditions concerned, the scale of inequalities is unlikely to change quickly, so these results using the 1998 survey will give us information about inequalities and provide a benchmark for considering how inequalities may change in the future. The greatest value of the data reported here relates to what they tell us about what we measure when examining inequalities in oral health.

The inequalities are much larger when measured using the number of teeth measure compared with OHIP. For the number of teeth, the Gini coefficient for the UK is 0.6769 including the edentate and 0.4039 when they are excluded.^1^ This compares to a Gini for overall health inequality of 0.1131 in England (20).^2^ The latter is based on a sample of the English population over 50 and will be lower because the variation in health in a restricted age group will be smaller, but the difference in these measures is still striking. In terms of income-related inequality, for the number of teeth, CI was 0.3534 (including edentate). This is also substantially higher than CIs for general health of 0.012 and 0.11 calculated by Jurges (20) and Van Doorslaer et al. (21), respectively.

The age-related increases in inequality for the number of teeth measure highlight how inequalities may operate across long periods of life, specifically the impairment (loss of teeth) resulting from cumulative damages due to dental caries, periodontal disease and their sequelae. An individual's number of teeth is affected by numerous factors across the lifetime including disease risks, personal behaviours, behaviours of the professionals (such as dentists) and upstream contextual determinants. The number of teeth therefore captures a lifetime cumulative experience. By contrast, the OHIP measure captures the way that a person's oral condition has impacted on their daily life in recent months.

These data could be identifying both important cohort and age effects. Older individuals will have come from a time when oral health outcomes were generally worse in the population as a whole (1,22). Consequently, we cannot assume that inequality of this scale will persist as time changes. Future generations may experience different distributions of oral health as a result of changing attitudes, technologies and behaviours, and this may alter the scale of inequality.

In many studies of inequality in general health, there are healthy survivor effects that can result in falling inequalities in older age. Using the number of teeth as the outcome measure, we see inequalities are substantially worse later in life, as individuals who have had poor oral health for a period of years start to see the long-term physical damage that eventually results. This is evident in both pure inequality and income-related inequality. Furthermore, inequalities are larger when edentate individuals are included in the analysis. Adding observations increases the variation, especially in this case where the edentate represents those individuals with the poorest oral health, and often the lowest incomes.

The results also highlight the difference between objective clinical and self-reported measures of health. There are stark differences in measured inequality, the determinants of health and the decomposition when comparing OHIP (the self-reported assessment of impact and related quality of life) to the number of teeth (an objective clinical measure). The relationship between age, tooth loss and OHIP is not straightforward. Epidemiological studies have demonstrated that younger groups often have worse OHIP despite having more teeth (23,24). Inequality is substantially less overall when measured by OHIP for the whole population, and the pattern related to age is also fundamentally different. In the younger age groups, when measured using the OHIP, inequality is generally larger than when measured using the number of teeth. In the older age groups (above 50 for the Gini coefficients and above 40 for the CI), this situation is reversed.

Earlier work, investigating the differences between objective and self-reported measures of health, concludes that self-reported measures are subject to measurement error, making them unreliable for investigating health inequalities (13,25).^3^ This may be the case but there may be further issues when considering self-reported measures, specifically the differences in expectations of health (which may be generational) and the capacity for an individual to adjust their expectations of health to their circumstances (which may be age related). In both cases, one might postulate that social conditions and personal contexts would be important in making an assessment of one's own health, unlike the objective measure of number of teeth which is a cold record of historic physical damage to the dentition.

The substantially lower levels of inequality in the OHIP may be related to either different expectations from older generations or adaptation to changed circumstances with increasing age. For policy makers, this raises an important but very awkward question: is it important to focus on the inequalities from objective measures, requiring a lifetime commitment, or to assume that more deprived individuals will accept their poor health situation, and concentrate on self-reported measures that represent the patients' own present perceptions of their oral health and not worry about the future? Furthermore, if there are cohort effects at work here, such adaptation may not automatically follow in future generations. Current older generations, who have a shared experience of poorer dental health, may be more willing or able to adapt than younger generations. Over time it is possible that we may observe the differences between clinical measures and measures of the impact of oral health becoming closer.

The decomposition results show that the determinants of income-related health inequality contribute differently to income-related inequality depending on the measures of oral health used, suggesting that different policy responses may be needed based on the oral health measure examined. It also has been shown that the distribution of the factors is important, suggesting that policy makers may need to try and change the distribution of the determinants. Another policy implication relates to the Marmot's recommendation on proportionate universalism. We see a gradient of inequalities across the material measure of income and also for other demographic factors such as age and economic activity status. Marmot (3) suggests that the existence of these gradients means that we should not solely focus on the most disadvantaged, but take universal actions with a scale and intensity that is proportionate to the level of disadvantage and in particular, also to the level of other demographic factors such as age.

The strength of the study lies in the ability to apply the more sophisticated inequality measures in the field of oral health and the opportunity to investigate both objective and self-reported measures of the impact of oral health with this unique and rich data set. Our findings have important implications for how we should measure health inequality when faced with different type of health indicators and how we should consider the cohort and age effects with changing populations' perceptions. There are, however, some limitations to this study. One of the main issues is the low explanatory power of the OHIP regression, which may indicate the existence of other unobservable factors that affect self-reported measure of oral health. Further research will look to address the limitations and conduct comparisons over time using future waves of the Adult Dental Health Surveys.

## Conclusion

We have demonstrated that oral health inequalities exist and this is consistent with earlier findings. We also find that inequalities depend on the outcome measure of oral health that is used as an outcome. Further, using sophisticated methods to measure inequality, we have been able to compare inequality in oral health with inequality in general health.

These results have highlighted a number of issues surrounding inequalities in oral health and the importance of income as a determinant of oral health, even in a country with a publicly funded oral healthcare system. The cumulative nature of the impairments that result from oral diseases and the way they are treated create very specific issues for measuring inequalities in oral health. Self-reported measures of impact may be affected by adaptation of the individual to any impairment and so may be quite dynamic. Clinical measures are costly to collect, may require lifelong commitment and do not take into account individuals' quality of life. This methodology issue is highlighted for future research.

## Footnotes

a The figures presented here are adjusted using Erreygers' method; however, even using unadjusted Gini indices, our results are still higher compared with Gini of the population's general health found in the literature.

b Making comparisons across the literature always has to be treated with caution because of different sample sizes and different adjustment methods (or in some studies including the Jurges (20) study, no adjustments are made).

c There is also a body of work using vignettes to investigate problems of heterogeneity in the reporting of self-assessed health (26–28).
